# Functional Biogeography as Evidence of Gene Transfer in Hypersaline Microbial Communities

**DOI:** 10.1371/journal.pone.0012919

**Published:** 2010-09-23

**Authors:** J. Jacob Parnell, Giovanni Rompato, Leigh C. Latta, Michael E. Pfrender, Joy D. Van Nostrand, Zhili He, Jizhong Zhou, Gary Andersen, Patti Champine, Balasubramanian Ganesan, Bart C. Weimer

**Affiliations:** 1 Center for Integrated BioSystems, Utah State University, Logan, Utah, United States of America; 2 Department of Biology, Utah State University, Logan, Utah, United States of America; 3 Department of Nutrition & Food Sciences, Utah State University, Logan, Utah, United States of America; 4 Ecology Center, Utah State University, Logan, Utah, United States of America; 5 Institute for Environmental Genomics, Department of Botany and Microbiology, University of Oklahoma, Norman, Oklahoma, United States of America; 6 Lawrence Berkeley National Laboratory, University of California, Berkeley, California, United States of America; Cairo University, Egypt

## Abstract

**Background:**

Horizontal gene transfer (HGT) plays a major role in speciation and evolution of bacteria and archaea by controlling gene distribution within an environment. However, information that links HGT to a natural community using relevant population-genetics parameters and spatial considerations is scarce. The Great Salt Lake (Utah, USA) provides an excellent model for studying HGT in the context of biogeography because it is a contiguous system with dispersal limitations due to a strong selective salinity gradient. We hypothesize that in spite of the barrier to phylogenetic dispersal, functional characteristics—in the form of HGT—expand beyond phylogenetic limitations due to selective pressure.

**Methodology and Results:**

To assay the functional genes and microorganisms throughout the GSL, we used a 16S rRNA oligonucleotide microarray (Phylochip) and a functional gene array (GeoChip) to measure biogeographic patterns of nine microbial communities. We found a significant difference in biogeography based on microarray analyses when comparing Sørensen similarity values for presence/absence of function and phylogeny (Student's t-test; p = 0.005).

**Conclusion and Significance:**

Biogeographic patterns exhibit behavior associated with horizontal gene transfer in that informational genes (16S rRNA) have a lower similarity than functional genes, and functional similarity is positively correlated with lake-wide selective pressure. Specifically, high concentrations of chromium throughout GSL correspond to an average similarity of chromium resistance genes that is 22% higher than taxonomic similarity. This suggests active HGT may be measured at the population level in microbial communities and these biogeographic patterns may serve as a model to study bacteria adaptation and speciation.

## Introduction

Change in community composition with distance, time, and along environmental gradients (β-diversity) provides information about the mechanisms that generate and regulate microbial biodiversity [1]–[7] and provide insight into evolutionary history [8] and ecosystem function [9]. Although community structure, evolution [10] and functional diversity [11] are all influenced by horizontal gene transfer (HGT), HGT is rarely linked to relevant population-genetics parameters and temporospatial considerations [12]. Genome sequence analyses indicate that preferential transfer of genes is strongly correlated with gene function and is a frequent process in microbial evolution [13] accounting for much of the biodiversity among isolates [14]–[15]. Genome sequence comparisons (nucleotide and dinucleotide frequency; [16], codon usage bias; [17]–[19], or Markov analyses; [20]–[21]) demonstrate horizontal gene transfer of individual organisms, however our current view of HGT is incomplete as it lacks blending population genetics, microbial ecology, and biogeography.

Measuring the transfer of functional genes within ecosystems and relating these events to environmental conditions is a substantial challenge [22]–[23]. Spatial distribution models have been applied successfully in microbial ecology [3]–[5], [9], [15], [24], in some cases shifting the focus of biogeography from the taxonomic level to functional characteristics that enable survival [4], [9]. This shift provides a foundation for detailed molecular-level analyses within the context of a sound ecological and evolutionary framework that is required for spatially determining the rate and extent of real world physical gene transfer [25]–[26]. To our knowledge, linking the spatial distribution of functional genes with environmental conditions in a contiguous system has never been addressed.

In this study we examined taxonomic and functional biogeography in the context of the selective pressures in the Great Salt Lake, Utah (GSL). GSL is a hypersaline environment where NaCl concentration ranges from near seawater to saturation, with exceptionally high concentrations of sulfate [27] and heavy metals [28] throughout the lake. We analyzed the microbial biodiversity and functional potential across nine sites, chosen for extremes in salt concentration, throughout GSL. Because the majority of environmental microbes cannot be cultured with current laboratory techniques, we utilized recent advances in environmental microarray technology to profile the community structure (using the PhyloChip microarray capable of identifying over 8,000 taxa; [29]) and functional gene characteristics (using the GeoChip microarray capable of identifying over 10,000 genes in 150 different functional groups; [30]).

## Materials and Methods

### Sampling strategy and environmental measurements

In the summer of 2007, 9 water samples were collected from different sites throughout GSL (Figure S1): Rozel Point (RP, salt saturated; 41°25′56.13″N 112°39′48.31″W), Antelope Island (AI, high salt; 41°02′22.37″N 112°16′42.33″W), Farmington Bay (FB moderate salt; 41°03′31.30″N 112°14′04.98″W), USGS site 3510 (35 40°51′11.07″N 112°20′33.11″W cords) in the South Arm at 3 depths (surface, 3510S; 15.3%, 7m, 3510I; 18%, and 9m, 3510DB; 20% salt concentration) and USGS site 2565 (25, 41°06′58.79″N 112°40′48.33″W) in the South Arm at 3 depths (surface 2565S; 15.4%, 7m; 2565I, 23.1%, and 9m, 2565DB; 23.2% salt concentration). Water from the lake sites was collected at various depths either directly (surface samples) or using a peristaltic pump with flexible tubing that was weighted to minimize horizontal drifting. Samples were collected in sterile Nalgene® 4L plastic bottles. Within 6 h of collection, samples were refrigerated at 4°C until processing. This sampling strategy provided points of data for community analysis ranging from near freshwater to salt-saturated brine.

In order to determine prevailing environmental conditions in which microbial communities reside, we measured dissolved oxygen, pH, salinity via electrical conductivity, and temperature. Water chemistry parameters were measured at lake sites during time of sampling using an In-Situ Troll 9500 multiparameter water-quality monitor. The high range specific conductance and standard pH probes were calibrated and verified prior to taking measurements. Additional measurements involving long-term environmental variation are available through USGS records for sites 3510 and 2565.

### Extraction of GSL Community DNA

We optimized protocols for the extraction of community DNA from the hypersaline waters of GSL using a modification of a protocol published by Griffiths *et al.* [31]. Due to the near-saturated salt concentration, bacteria cannot be isolated from the samples by filtration as salt precipitates clog the filter. As an alternative, one gallon of water collected from GSL was centrifuged (10,000×g, 40 min, 4°C) in a Sorval high speed centrifuge and resuspended in 500µl of modified CTAB (hexadecyltrimethylammonium bromide) extraction buffer (equal volumes of 10% CTAB in 0.7 M NaCl and 240 mM potassium phosphate buffer (pH 8) [32]. Commonly used bead-beating and chloroform procedures were employed to extract DNA [31]. The extracted community DNA was purified by passing it through a Sephacryl® S-300 column. Briefly, the column was constructed by plugging a 5 ml syringe with sterile glass wool, pouring 5 ml of resin suspended in 24% ethanol into the syringe and centrifuging 10 minutes at 1000×g at room temperature. The column was washed twice with sterile ddH_2_0. Samples were added to the column and purified by centrifugation for 10 minutes at 1,000×g at room temperature. We found that use of this column is critical for good resolution of community DNA and for the elimination of PCR inhibitors present in the water collected from GSL. With this protocol, we have successfully extracted archaeal and bacterial DNA from hypersaline environments, including GSL, and used this DNA to amplify 16S rRNA genes by PCR.

### Taxonomic diversity

To assess microbial diversity and to overcome obstacles of non-cultivability we used a newly developed 16S Phylogenetic Array (Phylochip) containing probes for 8,741 bacterial and archaeal taxa [29]. Hybridization of the PhyloChip is achieved using slightly modified Affymetrix (Santa Clara, CA) protocols (see ref. [29]). Briefly, the ribosomal 16S gene was amplified by PCR utilizing Bacteria (F: 5′-AGAGTTTGATCCTGGCTCAG-3′, R: 5′-ACGGCT ACCTTGTTAGCACTT-3′) or Archaea (F: 5′-GACGGGCGGTGTGTCA-3′, R: 5′-GCGGATCCGCGGCCGCTGCAGAYC-3′) specific primers. To minimize the primer bias, PCR amplification was performed with a temperature gradient from 48°C to 58°C for the annealing temperature. The PCR products from the different amplification reactions were collected, purified, and quantified. Two hundred ng of 16S amplicon were fragmented by DNaseI digestion for 20 minutes at 25°C. The DNaseI was then inactivated and the fragmented DNA was biotin labeled for 60 minutes at 37°C following the Affymetrix protocol. The labeled DNA was added to Affymetrix hybridization solution and hybridized to a PhyloChip for 16 hours at 48°C rotating at 60 rpm. The chip was washed and stained following the Affymetrix protocol and scanned utilizing an Affymetrix ChiScanner 3000. Intensity values were normalized using Robust Multi-Array normalization [33].

### Functional diversity

To determine the functional genomics capabilities of the microbial communities within GSL, we used the GeoChip functional gene array [30]. Extracted community DNA (no amplification step) was labeled with cyanine-5 (Cy-5) dye. Briefly, approximately 2 µg of genomic DNA was denatured for 5 min at 99.9°C in solution with random octamer mix (Invitrogen, Carlsbad, CA, USA) and snap chilled on ice. Following denaturation, 2.5mM dithiothreitol (DTT), 0.25mM dATP, dCTP and dGTP, 0.125mM dTTP, 0.125mM Cy5-dUTP, and 80U Klenow fragment (Invitrogen, Carlsbad, CA, USA) were added. Reaction mixtures were incubated at 37°C for 3 h. Labeled target DNA was purified with a QIAquick PCR kit (Qiagen, Valencia, CA, USA) according to the manufacturer's instructions. Labeled DNA was measured on a ND-1000 spectrophotometer (NanoDrop Technologies, Wilminton, DE) and dried using a speed-vac at 45°C for 45 min. Dried, labeled DNA was resuspended in a solution of 50% formamide, 5×sodium saline citrate, 0.1% sodium dodecyl sulfate, 0.1 µg µl^−1^ herring sperm DNA and 0.85 mM dithiothreitol and incubated at 95°C for 5 min. Labeled reactions were kept at 60°C until hybridization. Two technical replicates of community DNA hybridizations were performed using a HS4800 Hybridization Station (TECAN US, Durham, NC) and hybridization conditions were followed as indicated elsewhere [34] with hybridization temperature of 42°C. GeoChip microarrays were scanned using a ProScanArray microarray scanner (PerkinElmer, Boston, MA) as mentioned by Yergeau *et al.*, [34]. Scanned images were analyzed using ImaGene 6.0 software (BioDiscovery, El Segundo, CA, USA) with signals processed as signal to noise ratio >2.0. The phylogenetic and functional microarray data used in this study comply with journal standards and will be made freely available.

### Selective pressure

**Figure 1:**
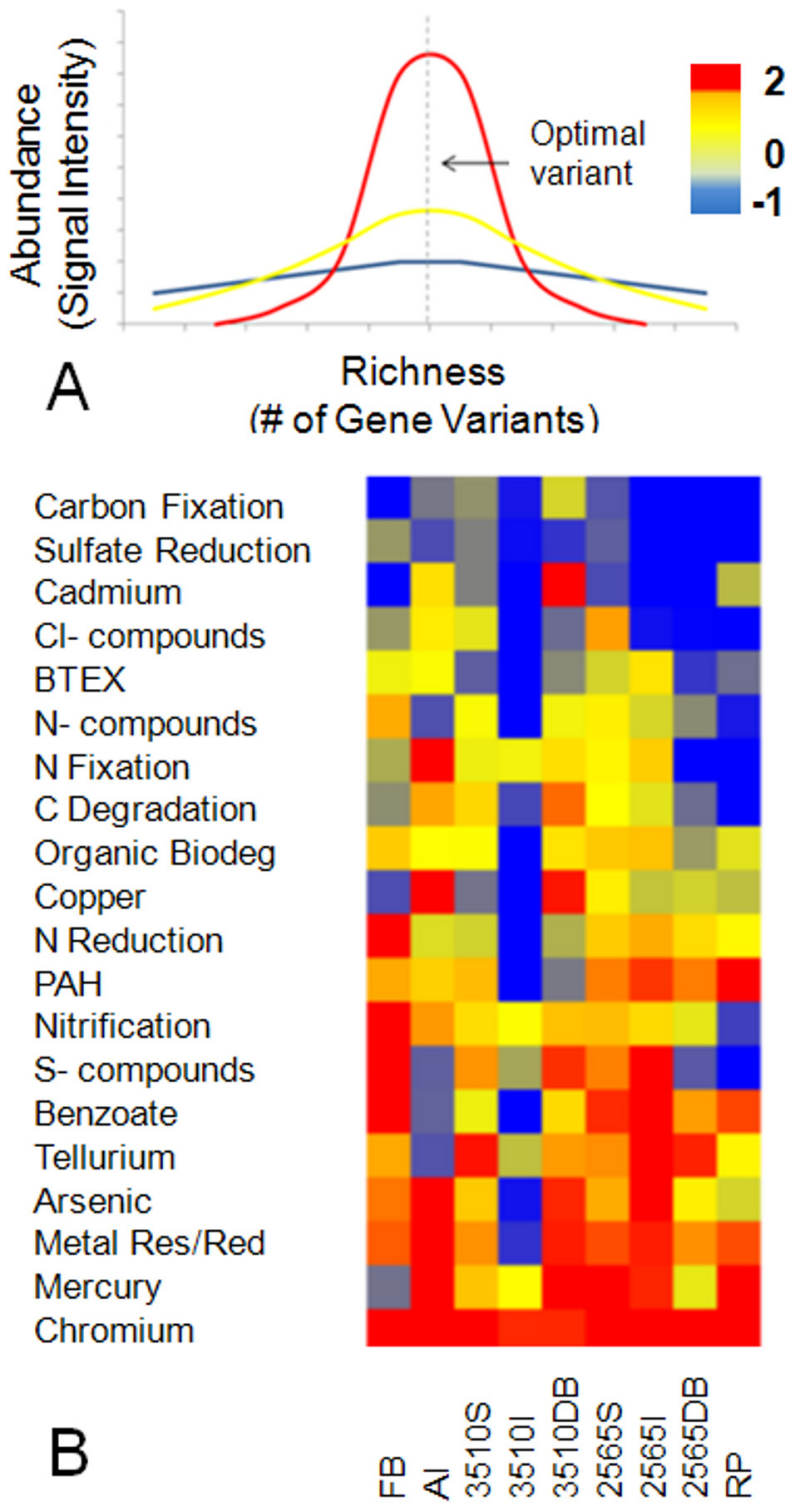
Selective pressure in Great Salt Lake (GSL). Model of selective pressure (A) indicates that high selection causes an increase in optimal gene variant(s) at the expense of inefficient gene groups resulting in a high abundance/richness ratio (red). Conversely, low selective pressure results in a broad range of diverse functional variants resulting in a low abundance/richness ratio (blue). Top right: color scale and numerical value for log_2_ transformed ratio of abundance/richness. Selective pressure of functional genes by location (B) calculated using functional gene array intensity values relative to gene variants (log_2_ transformed) show high selective pressure for heavy metals, particularly mercury, arsenic and chromium and low selective pressure for carbon fixation and sulfate reduction. Functional groups are ordered by the average abundance∶richness ratio throughout GSL. Heat map colors correspond to the type of abundance∶richness curve in (A) for each location (Antelope Island = AI, Farmington Bay = FB, sites 3510 Surface, Interface, and Deep Brine, sites 2565 Surface, Interface, and Deep Brine, and Rozel Point = RP) and each functional group (poly aromatic hydrocarbon = PAH, benzene, toluene, ethylbenzene, xylene,  = BTEX). Blue indicates low selective pressure, red indicates high selective pressure.

Selective pressure was determined by taking the intensity for different groups of functional genes considered relative to the number of gene variants detected in each group [34]. The microarray design contains multiple probes for each gene sequence or each group of homologous sequences. The richness of gene variants (different gene sequences with the same function) detected for each functional group provided evidence of functional redundancy within each spatially distinct community. Similarly, comparison of the Log_2_ normalized probe intensity for each functional category indicated the relative abundance of each gene. The relative number of gene variants was determined by dividing the number of genes belonging to each functional category by the total number of genes detected [34]. Relative intensity values for each hybridization signal were calculated and ranked according to intensity to allow comparison of relative abundance of genes in each functional category across experimental samples as per Yergeau, et al., (2007). Figure 1A illustrates the model distribution curve of functional genes through different levels of selective pressure using the competitive exclusion model.

### Beta diversity

Beta diversity estimates were calculated using presence/absence for individual genes grouped into functional categories as well as 16S genes. Because of the nature of the different arrays (phylochip is PCR-based), we restricted biogeographical analyses where direct comparisons were made to presence/absence based on normalized signal intensity for each array type. We used Sørensen's index for dissimilarity (Bray-Curtis or percent dissimilarity):
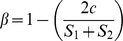
where, S_1_ = the total number of genes within a specific functional group detected in the first community, S_2_ = the total number of genes within a specific functional group detected in the second community, and c = the number of genes within a specific functional group common to both communities. The Sørensen index ranges from 0 to 1 where 1 indicates completely different communities and 0 indicates identical communities. Comparison of pairwise dissimilarity across GSL was performed using Mantel tests. To assess the significance of the observed number of shared functional genes between communities, and to test the null hypothesis of random assemblage of communities at sites, we resampled from the total functional gene set to construct 10,000 simulated data sets for each sampling site and estimated the number of shared genes in pair wise comparisons. Site-specific resampling was constrained by the total observed number of observed genes at each site. From these simulated data sets a distribution of shared genes for each pair wise comparison was used to generate significance levels for the observed overlap in functional gene sets.

## Results

### Environmental variability and microbial diversity

We detected over 5,000 different 16S rRNA gene sequences of diverse microbial taxa from 9 microbial communities analyzed from GSL ranging from approximately 100 community members in the salt-saturated brine of Rozel Point to 2,400 members in the deep brine sediments in the South Arm (sample site 2565). We detected over 4,500 different functional genes in GSL ranging from 227 different functional genes in the salt-saturated RP community to over 3,000 in the interface between the deep brine layer and surface waters (sample site 3510). The total number of functional genes did not correlate with taxonomic richness across all pooled samples (Pearson correlation, n = 9, r = 0.28), however the fluctuation in dissolved oxygen among South Arm sites (3510 and 2565) is positively correlated with the ratio of functional genes (GeoChip) to taxa (Phylochip) (Pearson correlation, p = 0.046, r = 0.82) (Table 1).

**Table 1:** General environmental parameters and α-diversity associated with sample sites.

		Annual Variation		α-Diversity		
Sample location	Salinity (%)	dO (mg/L)	Temp C°	Phylogenetic	Functional	Ratio
Farmington Bay	5	nd	nd	592	637	1.08
Antelope Island	15	nd	nd	317	1,994	6.29
Rozel Point	30	nd	nd	100	227	2.41
3510 Surface	15.3	3.47	7.67	1,724	1,167	0.68
3510 Interface	18	3.77	8.11	1,305	3,053	2.34
3510 Deep Brine	20	0.6	3.9	1,079	411	0.38
2565 Surface	15.4	3.77	7.8	914	2,383	2.61
2565 Interface	23.1	0.95	5.85	1,423	487	0.34
2565 Deep Brine	23.2	0.8	4.38	2,400	896	0.37

nd not determined.

### Selective pressure

Using an approach based on the competitive exclusion principle (see methods), we estimated the selective pressure for each functional category by analyzing the distribution of genes within each sample location. Figure 1B indicates the ratio of the relative intensity to relative richness for each functional group in each location examined throughout GSL. Although the ratio for most functions varies lake-wide, carbon fixation and sulfate reduction ratios are low in all locations, and chromium resistance ratios are high in all locations. Examples of curves for 3510 interface site chromium resistance and sulfate reduction genes are demonstrated in Figure S2.

### Taxonomic and functional biogeography throughout GSL

**Figure 2:**
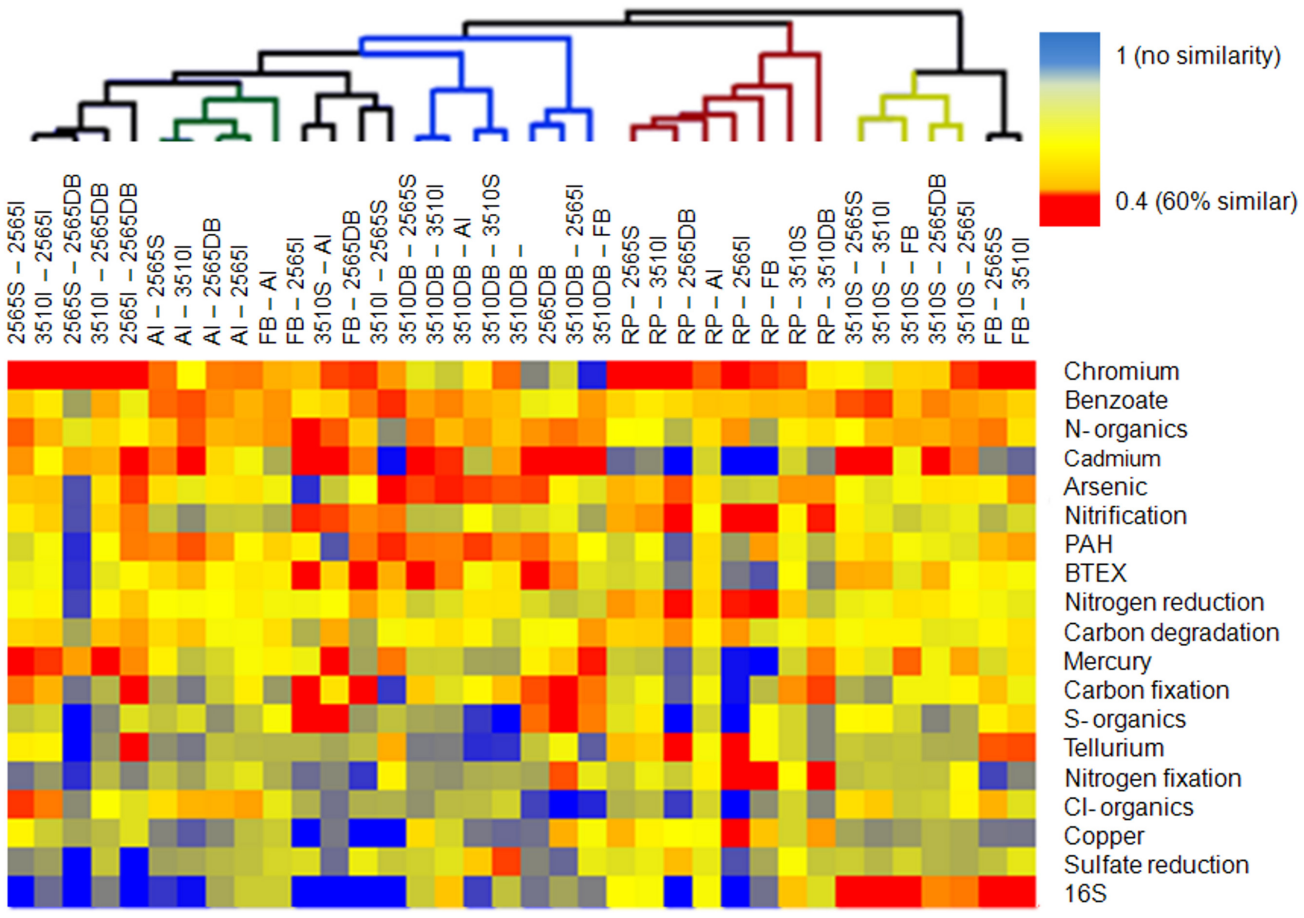
Difference in Sørensen similarity index between key functional genes (rows) and total function for paired sites (columns). Blue indicates less similar β-diversity index in relation to the average of all functional groups, red indicates more similar relative to all function (top right color scale). Rows are ordered by sum of similarity indices across Great Salt Lake. Columns are site-to-site comparisons and are clustered using Pearson's correlation coefficient UPGMA (Unweighted Pair Group Method with Arithmetic Mean).

We used Sørensen's β-diversity to delineate regions or transitions of functional genes (GeoChip) throughout GSL and compared these with taxonomic delineations determined using the PhyloChip. Figure 2 shows the pairwise comparison of the similarity matrix of sample locations for 16S rRNA genes as well as individual functional groups such as metal resistance genes (further divided by specific metals), organic biodegradation genes, and chromosomally encoded functions (sulfate reduction, carbon fixation, etc.) relative to all functional genes detected on the functional gene array. Based on randomized simulated data sets the observed similarity of functional genes between sites is significantly greater than expected by chance for all comparisons except those involving the Antelope Island site and the 3510S site (Table S1). Analysis of chromosomally encoded functions (including sulfate reduction genes) show low (not significant) similarity between sites (Mantel, r = 0.47, p = 0.11) while we found significant biogeographic patterning for metal resistance (Mantel, r = 0.53, p = 0.04). Comparison of the β-diversity indices for 16S and functional genes indicates that the change in taxonomic diversity and function is significantly different throughout GSL (pairwise Student's t-test, n = 36, p = 0.005; see Table S2). Sørensen's diversity in relation to geographic distance shows a very weak correlation in both taxonomic and functional genes (Figure S3).

**Figure 3:**
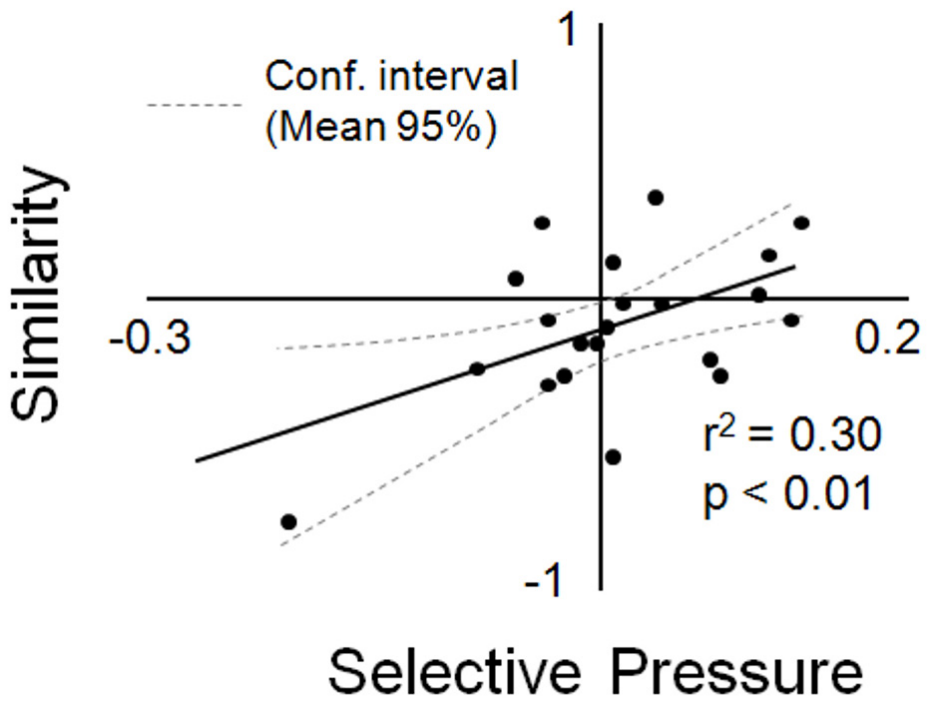
Correlation between total selective pressure (across the entire GSL) measured by competitive exclusion and Sørensen's β-diversity. β-diversity values are normalized to total function (similarity of specific function vs all function) and the values are Log_2_ transformed. Selective pressure is determined by the ratio of gene abundance to gene richness and Log_2_ normalized to total gene abundance∶richness. Data are shown as linear regression model.

Similarity values for each functional group were normalized to the similarity value for all functional genes and Log_2_ transformed. This provides information on which functional groups are more similar than others throughout the lake. Figure 3 demonstrates a weak, yet significant correlation between the relative intensity/richness value calculated above and similarity. Spatial variability of selective pressure across different sites (Figure 1B) breaks the premise of competitive exclusion and, as expected, lessens the correlation near the mean of similarity and selective pressure.

## Discussion

In general, higher salt conditions are restrictive to Cyanobacteria, β-proteobacteria, and Bacteroides, and favor Archaea and Thermotoga (Figure S4). We suggest that the variation in functional diversity within these communities may reflect the environmental dynamics associated with each location. Because of its direct link with the functional repertoire, the diversity of function in relation to the diversity of organisms is believed to be closely coupled to the functional complexity and environmental niche of an organism [35], [36]. Unvarying environmental conditions favor organisms with a narrow functional repertoire of genes (specialists) while variable environmental conditions favor versatile organisms (generalists) with a wide range of functional potential [23]. Ratios of gene richness to phylogenetic richness in two long-term sites (six samples) when compared with USGS abiotic measurements suggest that more versatile organisms (larger relative functional diversity) are found in areas that vary greatly in oxygen concentration (Table 1). Although variations in oxygen are not responsible for driving all genetic diversity, these data suggest that environmental pressures drive functional diversity in GSL and are consistent with metagenome analyses of HGT [37]. Consequently, the distribution and frequency of functional genes throughout different communities provide insight to environmental pressures experienced by these microbial consortia.

The functional gene array provides a powerful tool for studying microbial biogeography [9] and ecosystem dynamics in various environments [38]. The functional gene array has sufficient resolution at the functional level to demonstrate how changes in environmental conditions affect the functional structure of microbial communities [39]. In addition, it offers some predictive value with respect to estimating enzymatic activities in microbial communities related to gene families, making correlations between gene abundances and ecological significance rather straightforward [34]. Moreover, the number of gene variants detected offers insight to possible functional redundancy among the dominant community members, while absolute hybridization intensity is indicative of relative abundance of genes [34].

Resource limitation often drives selection through competitive exclusion [40] where groups more adept at acquisition and more efficient at resource utilization excel, resulting in fewer competitors (as inefficient competitors decline) (Figure 1A). Similarly, the frequency of a specific function reflects its relative importance in an environment [24] and is used here as an indicator of selective pressure and successful competition. The principle of competitive exclusion is apt here as the conditions of a single limiting resource (substrate) and as assumption of spatially independent communities. As selective pressure increases, the functional redundancy throughout the community declines with an increase in abundance of functionally similar and competitive variants. For example, high concentrations of chromium throughout GSL [27] provide a selective advantage for organisms containing the most effective chromium resistance strategies. These more efficient mechanisms increase within the population (either as resistant organisms multiply or as genes are duplicated in the population) and ineffective resistance mechanisms disappear due to toxicity of the environment. The ratio of the relative intensity to relative richness (Figure 1B), therefore, provides a metric for the selective pressure throughout GSL. Conversely, the absence of selective pressure allows diversification of genes as less efficient variants pose no threat to fitness. Sulfate concentration in the GSL is extremely high and is not likely a limiting factor in microbial growth [27]. Consequently, there is little selective pressure for more efficient sulfate reduction genes resulting in more variants and no dominant variants. In this case, the relative intensity is low whereas the number of gene variants is high (Figure 1B). Variation in function, presumably via HGT, rather than changing community, is controlling gene distribution within the environment. Beta-diversity describes the change in biodiversity over space, time, or environmental gradients and often provides ecological and evolutionary information on dispersal, speciation processes, and species turnover. Generally, beta-diversity is used to quantify the species change or turnover in order to delineate biotic regions or transitions [4]. In the case of this study, we use beta-diversity (dissimilarity) to quantify the spatial change of functional genes within the environment. Biodiversity studies are often hampered by artifacts associated with sampling [3], [4] which in this case is minimized using array technology. Each array contains probes for about ten thousand genes, and hence a single hybridization can simultaneously survey a good portion of microbial populations [9]. Despite being a closed format that provides information only about the genes present on the microarray, the Phylochip and GeoChip ensure unbiased comparison of microbial communities because each community is tested against the same set of probes [9]. Although the scale makes a difference in conclusions based on biodiversity estimates [41], both arrays used here are based on the gene-level scale.

**Figure 4:**
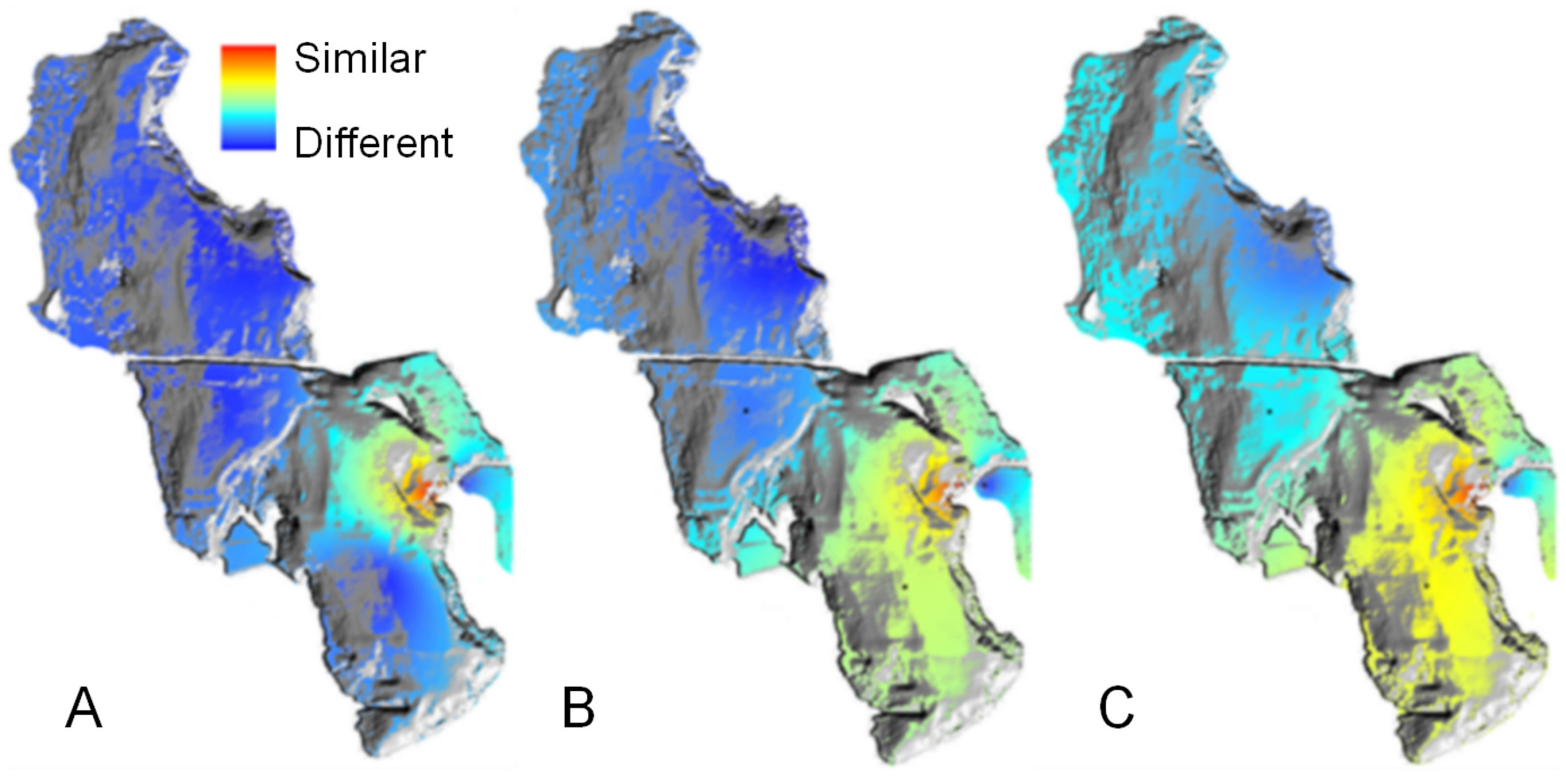
Biogeographic similarity across GSL. Taxanomic distance decay (16S) (A), sulfate reduction (B), and chromium resistance (C). All similarity values are relative to the community of the Antelope Island sample. Similarity values are mapped using inverse distance weighted interpolations analysis and overlaid on a bathymetric map of GSL using ArcGIS.

In order to treat the two different approaches (one PCR based, one not) cautiously, we looked at the presence/absence for genes and community members. The average similarity decay of 16S rRNA genes is low throughout GSL (Figure 4A), translating into dispersal limitations presumably due to the salinity gradient. The similarity of all functional genes is significantly higher than that of 16S genes, indicating higher dispersal for all functional gene groups analyzed. These observations are comparable with studies that show a difference in the historical rate of gene transfer between informational genes (16S) and operational genes (function) [12]. Within functional groups, the extent of gene transfer is dependent on whether the function is part of the microbial mobilome [25] or whether it is chromosomally encoded as part of the core genome [42] with the exception of phage-transferred genes [43]. Consequently, methanogenesis, a function that is known only to exist in Euryarchaeota (i.e. phylogenetically linked) shows similar biogeographic patterns to 16S genes throughout GSL (t-test p = 0.46; Table S1); this pattern is significantly different compared to chromium resistance patterns (Figure S5). This suggests that diversity patterns between the two different types of arrays are comparable and that biogeographic patterns of genes are not random nor are they a result of poor representation on the arrays used. The similarity between chromosomally-encoded sulfate reduction [44] genes across GSL is low, only slightly (6%) higher than the taxonomic similarity throughout GSL (Figure 4B), whereas similarity of plasmid/transposon-based chromium resistance genes [45] is 22% higher than the taxonomic similarity (Figure 4C). Although more intensive sampling would improve the resolution (see Figure 4), a significant difference in biogeographic patterns is evident.

We compared individual gene variants with their source to determine whether functional gene biogeography is cryptic within taxonomic biogeography or if the presence of highly dominant species would skew the comparison between taxonomic diversity and functional diversity. The chromium resistant gene sequenced from *Deinococcus radiodurans* R1 was the only chromium resistance gene detected in all samples from GSL; however, no 16S genes corresponding to any member of Deinococcus-Thermus group were detected in 2 of these samples. This suggests that although the chromium resistance genes likely originated from Deinococcus, they correspond to a different group possibly through a transfer event. Additionally, the most dominant chromium resistance genes throughout GSL corresponded to sequences from β-proteobacteria and α-proteobacteria despite inhibition of β-proteobacteria growth by salt [46]. These data suggest dispersal of functional genes that is independent of taxonomic biogeography.

### Conclusions and implications for HGT

The exchange of genetic material by microorganisms carries important implications for ecology, evolution, biotechnology, and medicine. HGT is an important factor in the evolution of prokaryotes in promoting adaptation to novel environments by allowing the exchange of large amounts of genetic information that increases the fitness of a specific population within an ecological niche [47]–[48] and plays a large role in controlling gene distribution within an environment by controlling the growth of specific populations [22]. The maintenance and dispersal of genetic elements depends on natural selection parameters that change spatially throughout GSL. Although the biogeographic patterns in GSL alone are not enough to imply HGT, the correlation of these patterns with selective pressure and mobility of functional genes (plasmid/transposon vs. chromosomal) throughout these microbial communities suggest that these patterns are not random. Consistent with previous observation [12], informational genes involved in transcription and translation, such as 16S, exhibit biogeographic patterns indicating very low levels of transfer compared with functional genes. Within functional genes, horizontal gene transfer corresponds to selective pressure. While gene transfer may occur frequently at the cellular level, this study provides the first demonstration of a measurable link between selective pressure and functional biogeography in a natural community and presents a valuable model for tracking and predicting the dispersal of microbial function.

In many cases increased similarity between sites corresponds to higher selective pressure (e.g. chromium) while decreased similarity corresponds to low selective pressure (e.g. sulfate reduction). Although this study is limited due to array-based analyses, similar approaches to metagenome sequencing datasets could provide improved understanding of the frequency and geographic extent of HGT in real-world communities.

## Supporting Information

Table S1 In A the observed number of functional genes in each site are shown in the diagonal and the observed overlap is shown in offdiagonal elements. The associated p-values are shown in B. The p-value is based on a distribution of shared genes generated from 10,000 simulated data sets sampling the observed number of functional genes in each community from the total set of 4560 genes and is the probability of the observed overlap given the null hypothesis of random asemblage of site-specific communities.
(0.16 MB PDF)

Table S2 Site-to-site Sørensen dissimilarity values according to functional groups.
(0.09 MB PDF)

Figure S1 Sample locations along the salinity gradient in Great Salt Lake. Sample sites 3510 and 2565 are USGS collection sites and samples were collected at the surface, deep brine layer, and the interface between surface and deep brine.
(1.01 MB TIF)

Figure S2 Example of curves from functional genes in 3510 surface sample used to determine selective pressure.
(0.18 MB TIF)

Figure S3 Weak correlation between dissimilarity and geographic distance. Circles represent taxonomic genes (solid line is linear regression). Cross hatches represent functional genes (dashed line is linear regression).
(0.14 MB TIF)

Figure S4 Major phylogenetic shifts due to increased salt. Farmington Bay (FB) was used as reference and the Log_2_ difference in intensity values are averaged (error = standard deviation) to indicate significant shifts due to high salt.
(0.11 MB TIF)

Figure S5 Average similarity of different genes throughout Great Salt Lake. 16S rDNA (phylochip)gene similarity is not significantly different from taxonomic-dependent methane generation (GeoChip). Sulfate reduction (low selective pressure) is not significantly different in lake-wide similarity from taxonomic genes. Chromium (high selective pressure) biogeographic patterns are significantly different, suggesting independence from taxonomy (t-test).
(0.23 MB TIF)

## References

[1] Achtman M, Wagner M (2008). Microbial diversity and the genetic nature of microbial species.. Nat Rev Microbiol.

[2] Fierer N, Jackson RB (2006). The diversity and biogeography of soil bacterial communities.. Proc Natl Acad Sci U S A.

[3] Green JL, Bohannan BJM (2006). Spatial scaling of microbial biodiversity.. Trends Ecol Evol.

[4] Green JL, Bohannan BJM, Whitaker RJW (2008). Microbial biogeography: from taxonomy to traits.. Science.

[5] Horner-Devine MC, Lage M, Hughes JB, Bohannan BJM (2004). A taxa-area relationship for bacteria.. Nature.

[6] Lozupone CA, Knight R (2007). Global patterns in bacterial diversity.. Proc Natl Acad Sci U S A.

[7] Strom SL (2008). Microbial ecology of ocean biogeochemistry: a community
perspective.. Science.

[8] Slater FR, Bailey MJ, Tett AJ, Turner SL (2008). Progress towards understanding the fate of plasmids in bacterial
communities.. FEMS Microbiol Ecol.

[9] Zhou J, Kang S, Schadt CW, Garten CT (2008). Spatial scaling of functional gene diversity across various
microbial taxa.. Proc Natl Acad Sci U S A.

[10] Brown JR (2003). Ancient horizontal gene transfer.. Nat Rev Genetics.

[11] Pal C, Papp B, Lercher MJ (2005). Adaptive evolution of bacterial metabolic networks by horizontal
gene transfer.. Nat Genetics.

[12] Thomas CM, Nielsen KM (2005). Mechanisms of, and barriers to, horizontal gene transfer between
bacteria.. Nat Rev Microbiol.

[13] Gogarten JP, Doolittle WF, Lawrence JG (2002). Prokaryotic evolution in light of gene transfer.. Mol Bio Evol.

[14] Feil EJ, Holmes EC, Bessen DE, Chan MS, Day NP (2001). Recombination within natural populations of pathogenic bacteria:
short-term empirical estimates and long-term phylogenetic consequences.. Proc Natl Acad Sci U S A.

[15] Lo I, Denef VJ, Verberkmoes NC, Shah MB, Goltsman D (2007). Strain-resolved community proteomics reveals recombining genomes
of acidophilic bacteria.. Nature.

[16] Xia X, Wei T, Xie Z, Danchin A (2002). Genomic changes in nucleotide and dinucleotide frequencies in
*Pasteurella multocida* cultured under high temperature.. Genetics.

[17] Klosterand M, Tang C (2008). SCUMBLE: a method for systematic and accurate detection of codon
usage bias by maximum likelihood estimation.. Nucleic Acids Res.

[18] Puigbo P, Guzman E, Romeu A, Garcie-Vallve S (2007). OPTIMIZER: a web server for opotimizing the codon usage of DNA
sequences.. Nucleic Acids Res.

[19] Putoni C, Luo Y, Katili C, Chumakov S, Fox GE (2006). A computational tool for the genomic identification of regions of
unusual compositional properties and its utilization in the detection of
horizontally transferred sequences.. Mol Biol Evol.

[20] Azadand RK, Lawrence JG (2007). Detecting laterally transferred genes: use of entropic clustering
methods and genome position.. Nucleic Acids Res.

[21] Tsirigos A, Rigoutsos I (2005). A new computational method for the detection of horizontal gene
transfer events.. Nucleic Acids Res.

[22] Smets BF, Barkay T (2005). Horizontal gene transfer: perspectives at a crossroads of
scientific disciplines.. Nat Rev Microbiol.

[23] Thomas CM, Nielsen KM (2005). Mechanisms of, and barriers to, horizontal gene transfer between
bacteria.. Nat Rev Microbiol.

[24] Dinsdale EA, Edwares RA, Hall D, Angly F, Briebart M (2008). Functional metagenomic profiling of nine biomes.. Nature.

[25] Frost LS, Leplae R, Summers AO, Toussaint A (2005). Mobile genetic elements: the agents of open source evolution.. Nat Rev Microbiol.

[26] Kassen R, Rainey PB (2004). The ecology and genetics of microbial diversity.. Annu Rev Microbiol.

[27] Brandt KK, Vester F, Jensen AN, Ingvorsen K (2001). Sulfate reduction dynamics and enumeration of sulfate-reducing
bacteria in hypersaline sediments of the Great Salt Lake (Utah, USA).. Microb Ecol.

[28] Naftz D, Angeroth C, Kenney T, Waddell B, Darnall N (2008). Anthropogenic influences on the input and biogeochemical cycling
of nutrients and mercury in Great Salt Lake, Utah, USA.. Appl Geochem.

[29] Brodie EL, DeSantis TZ, Moeberg Parker JJ, Zubietta IX, Piceno YM (2007). Urban aerosols harbor diverse and dynamic bacterial populations.. Proc Natl Acad Sci U S A.

[30] He Z, Gentry TJ, Schadt CW, Wu L, Leibich J (2007). GeoChip: A comprehensive microarray for investigating
biogeochemical, ecological, and environmental processes.. ISME J.

[31] Griffiths RI, Whiteley AS, O'Donnell AG, Bailey MJ (2000). Rapid method for coextraction of DNA and RNA from natural
environments for analysis of ribosomal DNA- and rRNA-based microbial
community composition.. Appl Environ Microbiol.

[32] Zhou J, Bruns MA, Tiedje JM (1996). DNA recovery from soils of diverse composition.. Appl Environ Microbiol.

[33] Irizarry RA, Bolstad BM, Collin F, Cope LM, Hobbs B (2003). Summaries of Affymetrix GeneChip probe level data.. Nucleic Acids Res.

[34] Yergeau E, Kang S, He Z, Zhou J, Kowalchuk GA (2007). Functional microarray analysis of nitrogen and carbon cycling
genes across an Antarctic latitudinal transect.. ISME J.

[35] Raes J, O Korbel J, Lercher MJ, von Mering C, Bork P (2007). Prediction of effective genome size in metagenomic samples.. Genome Biol.

[36] Bentley SD, Parkhill J (2004). Comparative genomic structure of prokaryotes.. Annu Rev Genet.

[37] Tamames J, Moyá A (2008). Estimating the extent of horizontal gene transfer in metagenomic
sequences.. BMC Genomics.

[38] Wang F, Zhou H, Meng J, Peng Z, Jiang L (2009). GeoChip-based analysis of metabolic diversity of microbial
communities at the Juan de Fuca Ridge hydrothermal vent.. Proc Natl Acad Sci U S A.

[39] Van Nostrand JD, Wu WM, Wu L, Deng Y, Carley J (2009). GeoChip-based analysis of functional microbial communities during
the reoxidation of a bioreduced uranium-contaminated aquifer.. Environ Microbiol.

[40] Hardin G (1960). The competitive exclusion principle.. Science.

[41] Parnell JJ, Crowl TA, Weimer BC, Pfrender ME (2009). Biodiversity in microbial communities: system scale patterns and
mechanisms.. Mol Ecol.

[42] Lefebure T, Stanhope MJ (2007). Evolution of the core and pan-genome of Streptococcus: positive
selection, recombination, and genome composition.. Genome Biol.

[43] Sullivan MB, Lindell DL, Lee JA, Thompson L, Bielawski JP (2006). Prevalence and evolution of core photosystem II genes in marine
cyanobacterial viruses and their hosts.. PLoS Biology.

[44] Lonegran DJ, Jenter HL, Coates JD, Phillips EJP, Schmidt TM (1996). Phylogenetic analysis of dissimilatory Fe(III)-reducing bacteria.. Appl Environ Microbiol.

[45] Branco R, Chung AP, Johnston T, Gurel V, Morais P (2008). The chromate-inducible *chrBACF* operon from the
transposable element Tn*OtChr* confers resistance to
chromium(VI) and superoxide.. J Bacteriol.

[46] Wu QL, Zwart G, Schauer M, Kamst-van Agterveld MP, Hahn MW (2006). Bacterioplankton community composition along a salinity gradient
of sixteen high-mountain lakes located on the Tibetan Plateua, China.. Appl Environ Microbiol.

[47] Kurland CG, Canback B, Berg OG (2003). Horizontal gene transfer: A critical view.. Proc Natl Acad Sci U S A.

[48] Ochman H, Lawrence JG, Groisman EA (2000). Lateral gene transfer and the nature of bacterial innovation.. Nature.

