# Supplementary material for: Functional Biogeography as Evidence of Gene Transfer in Hypersaline Microbial Communities
Source: PLoS One. 2010 Sep 23;5(9):e12919. doi: 10.1371/journal.pone.0012919 (PMC2950788; doi:10.1371/journal.pone.0012919)
Supplement: Table S1 — In A the observed number of functional genes in each site are shown in the diagonal and the observed overlap is shown in offdiagonal elements. The associated p-values are shown in B. The p-value is based on a distribution of shared genes generated from 10,000 simulated data sets sampling the observed number of functional genes in each community from the total set of 4560 genes and is the probability of the observed overlap given the null hypothesis of random asemblage of site-specific communities. (0.16 MB PDF) [file pone.0012919.s001.pdf]

Supplementary Table 1. In A the observed number of functional genes in each site are shown in the diagonal and the observed overlap is shown in offdiagonal elements. The associated p-values are shown in B. The p-value is based on a distribution of shared genes generated from 10,000 simulated data sets sampling the observed number of functional genes in each community from the total set of 4560 genes and is the probability of the observed overlap given the null hypothesis of random asemblage of site-specific communities.

A) Observed overlap in functional genes

	2565_Deep_Brine	2565_Interface	2565_Surface	3510_Interface	Rozel_Point	Antelope_Island	Farmington_Bay	Deep_Brine1	3510_Surface
2565_Deep_Brine	**896**	409	855	858	192	236	390	206	141
2565_Interface		**487**	463	468	167	145	271	114	80
2565_Surface			**2383**	2217	212	581	576	305	342
3510_Interface				**3053**	215	766	610	346	450
Rozel_Point					**227**	65	149	43	41
Antelope_Island						**1994**	186	299	986
Farmington_Bay							**637**	157	102
Deep_Brine1								**411**	213
3510_Surface									**1167**

B) Probability of observed overlap based on 10,000 random resamples

	2565_Deep_Brine	2565_Interface	2565_Surface	3510_Interface	Rozel_Point	Antelope_Island	Farmington_Bay	Deep_Brine1	3510_Surface
2565_Deep_Brine	-	p<0.001	p<0.001	p<0.001	p<0.001	p>0.999	p<0.001	p<0.001	p>0.999
2565_Interface		-	p<0.001	p<0.001	p<0.001	p>0.999	p<0.001	p<0.001	p>0.999
2565_Surface			-	p<0.001	p<0.001	p>0.999	p<0.001	p<0.001	p>0.999
3510_Interface				-	p<0.001	p>0.999	p<0.001	p<0.001	p>0.999
Rozel_Point					-	p>0.999	p<0.001	p<0.001	p=0.007
Antelope_Island						-	p>0.999	p<0.001	p<0.001
Farmington_Bay							-	p<0.001	p>0.999
Deep_Brine1								-	p<0.001
3510_Surface									-
