# Supplementary material for: Functional Biogeography as Evidence of Gene Transfer in Hypersaline Microbial Communities
Source: PLoS One. 2010 Sep 23;5(9):e12919. doi: 10.1371/journal.pone.0012919 (PMC2950788; doi:10.1371/journal.pone.0012919)
Supplement: Table S2 — Site-to-site Sørensen dissimilarity values according to functional groups. (0.09 MB PDF) [file pone.0012919.s002.pdf]

Supplementary Table 2. Site-to-site Sørensen dissimilarity values according to functional groups.

	16S (Phylochip)	Function (Geochip)	Core (no metals or organic pollutants)
2565I-2565DB	0.717	0.409	0.410
2565S-2565DB	0.936	0.404	0.498
3510I-2565DB	0.658	0.565	0.574
RP-2565DB	0.981	0.658	0.611
AI-2565DB	0.932	0.837	0.854
FB-2565DB	0.949	0.491	0.507
3510DB-2565DB	0.808	0.685	0.713
3510S-2565DB	0.803	0.863	0.518
2565S-2565I	0.941	0.677	0.700
3510I-2565I	0.875	0.736	0.745
RP-2565I	0.989	0.532	0.492
AI-2565I	0.947	0.883	0.896
FB-2565I	0.963	0.518	0.522
3510DB-2565I	0.835	0.746	0.759
3510S-2565I	0.831	0.903	0.918
3510I-2565S	0.441	0.184	0.182
RP-2565S	0.847	0.838	0.830
AI-2565S	0.927	0.735	0.765
FB-2565S	0.353	0.619	0.662
3510DB-2565S	0.839	0.782	0.825
3510S-2565S	0.486	0.807	0.839
RP-3510I	0.886	0.869	0.855
AI-3510I	0.920	0.696	0.737
FB-3510I	0.547	0.669	0.699
3510DB-3510I	0.779	0.800	0.839
3510S-3510I	0.537	0.787	0.822
AI-RP	0.961	0.941	0.949
FB-RP	0.776	0.655	0.611
3510DB-RP	0.910	0.865	0.865
3510S-RP	0.914	0.941	0.949
FB-AI	0.921	0.859	0.891
3510DB-AI	0.937	0.751	0.760
3510S-AI	0.931	0.376	0.372
3510DB-FB	0.817	0.700	0.713
3510S-FB	0.620	0.887	0.907
3510S-3510DB	0.792	0.730	0.743

Cl- compounds	N- compounds	BTEX	Benzoate	S- compounds	PAH	Other Organic	Tellurium	Mercury
0.429	0.411	0.412	0.422	0.429	0.377	0.595	0.294	0.381
0.465	0.421	0.511	0.454	0.544	0.516	0.495	0.563	0.383
0.574	0.556	0.589	0.539	0.652	0.574	0.564	0.642	0.461
0.826	0.721	0.769	0.650	0.882	0.796	0.859	0.481	0.800
0.802	0.804	0.844	0.784	0.957	0.796	0.850	0.908	0.886
0.543	0.480	0.368	0.452	0.556	0.452	0.530	0.535	0.550
0.852	0.643	0.556	0.707	0.625	0.623	0.567	0.739	0.688
0.922	0.826	0.831	0.801	1.000	0.891	0.844	0.955	0.887
0.594	0.613	0.700	0.659	0.731	0.718	0.798	0.679	0.549
0.678	0.708	0.753	0.734	0.781	0.751	0.821	0.740	0.646
0.750	0.500	0.625	0.518	0.833	0.600	0.742	0.429	0.700
0.838	0.844	0.881	0.848	0.952	0.891	0.900	0.966	0.900
0.571	0.419	0.429	0.515	0.273	0.519	0.627	0.568	0.533
1.000	0.683	0.692	0.764	0.455	0.720	0.719	0.765	0.727
0.909	0.847	0.918	0.855	1.000	0.945	0.912	1.000	0.860
0.203	0.212	0.176	0.160	0.200	0.160	0.209	0.176	0.169
0.923	0.849	0.889	0.823	0.875	0.892	0.903	0.811	0.898
0.727	0.713	0.724	0.668	0.795	0.684	0.707	0.839	0.737
0.594	0.570	0.619	0.592	0.621	0.597	0.541	0.556	0.652
0.893	0.717	0.659	0.738	0.872	0.720	0.728	0.914	0.836
0.800	0.816	0.771	0.722	0.815	0.803	0.774	0.868	0.805
0.947	0.882	0.910	0.867	0.867	0.885	0.928	0.849	0.940
0.669	0.629	0.671	0.623	0.733	0.624	0.670	0.817	0.757
0.701	0.664	0.683	0.657	0.657	0.635	0.609	0.596	0.662
0.873	0.772	0.737	0.743	0.898	0.740	0.718	0.933	0.855
0.767	0.758	0.754	0.689	0.792	0.770	0.751	0.855	0.822
1.000	0.933	0.932	0.915	1.000	0.931	0.959	0.962	1.000
0.750	0.731	0.800	0.631	0.667	0.619	0.742	0.667	0.889
1.000	0.857	1.000	0.827	1.000	0.947	0.864	1.000	0.800
1.000	0.943	0.951	0.910	1.000	0.967	0.960	1.000	1.000
0.892	0.806	0.859	0.806	0.875	0.843	0.801	0.941	0.862
0.818	0.707	0.768	0.721	0.946	0.662	0.746	0.958	0.840
0.444	0.339	0.370	0.370	0.273	0.459	0.383	0.420	0.296
0.900	0.656	0.733	0.662	0.647	0.710	0.636	0.846	0.600
0.955	0.845	0.925	0.859	0.943	0.906	0.834	0.957	0.805
0.778	0.710	0.725	0.707	1.000	0.679	0.685	0.926	0.818

Copper	Chromium	Cadmium	Arsenic	Other Metals	Sulfate reduction	C cycling	C Deg	C Fix
0.429	0.300	0.333	0.362	0.360	0.569	0.377	0.388	0.333
0.451	0.219	0.385	0.495	0.491	0.560	0.456	0.451	0.477
0.616	0.385	0.543	0.561	0.563	0.611	0.563	0.548	0.622
0.667	0.389	1.000	0.590	0.667	0.636	0.648	0.615	0.793
0.966	0.774	0.826	0.833	0.892	0.916	0.863	0.855	0.897
0.714	0.429	0.455	0.500	0.500	0.508	0.519	0.547	0.400
0.810	0.793	0.429	0.608	0.818	0.793	0.681	0.699	0.611
0.947	0.846	0.714	0.859	0.957	0.947	0.896	0.898	0.887
0.682	0.444	0.636	0.660	0.640	0.783	0.656	0.666	0.620
0.788	0.588	0.742	0.714	0.661	0.829	0.717	0.719	0.706
0.273	0.231	1.000	0.571	0.692	0.652	0.538	0.500	0.700
0.961	0.814	0.905	0.876	0.913	0.959	0.898	0.901	0.884
0.714	0.500	0.429	0.659	0.565	0.545	0.541	0.571	0.419
0.714	0.789	0.400	0.750	0.765	0.892	0.732	0.760	0.630
1.000	0.793	0.833	0.900	0.952	1.000	0.931	0.937	0.909
0.271	0.174	0.244	0.148	0.191	0.199	0.197	0.188	0.232
0.805	0.560	1.000	0.804	0.860	0.878	0.834	0.822	0.884
0.852	0.672	0.679	0.727	0.798	0.825	0.743	0.726	0.814
0.727	0.464	0.714	0.619	0.547	0.671	0.618	0.622	0.600
0.773	0.814	0.583	0.692	0.872	0.834	0.780	0.785	0.763
0.902	0.811	0.632	0.823	0.806	0.865	0.837	0.824	0.892
0.873	0.688	1.000	0.846	0.885	0.898	0.859	0.851	0.891
0.845	0.704	0.569	0.723	0.759	0.776	0.704	0.676	0.816
0.788	0.543	0.800	0.624	0.613	0.697	0.669	0.663	0.689
0.848	0.860	0.697	0.690	0.893	0.836	0.803	0.794	0.838
0.904	0.821	0.617	0.824	0.827	0.862	0.811	0.790	0.897
0.958	0.846	1.000	0.933	0.968	0.978	0.949	0.945	0.966
0.636	0.571	1.000	0.697	0.750	0.730	0.692	0.688	0.714
0.818	0.867	1.000	0.813	0.800	0.933	0.839	0.857	0.765
1.000	0.840	1.000	0.885	0.943	0.958	0.959	0.962	0.882
0.922	0.822	0.951	0.882	0.806	0.911	0.874	0.851	0.971
0.882	0.750	0.818	0.663	0.788	0.733	0.756	0.756	0.758
0.441	0.333	0.310	0.405	0.363	0.447	0.362	0.359	0.373
0.714	0.905	0.556	0.733	0.700	0.725	0.657	0.661	0.643
1.000	0.871	0.913	0.908	0.822	0.942	0.894	0.891	0.911
0.871	0.667	0.692	0.656	0.692	0.645	0.769	0.783	0.707

Methane oxidation	Methane generation	N Cycling	Nred	Nitrification	N Fixation
0.286	0.556	0.401	0.397	0.376	0.474
0.433	0.586	0.501	0.501	0.496	0.514
0.485	0.667	0.572	0.569	0.555	0.618
0.440	1.000	0.568	0.559	0.525	0.706
0.625	0.818	0.875	0.849	0.902	0.891
0.313	0.750	0.485	0.465	0.458	0.619
0.813	0.600	0.728	0.723	0.722	0.765
0.800	0.929	0.889	0.860	0.907	0.934
0.630	0.769	0.702	0.691	0.675	0.802
0.633	0.778	0.749	0.739	0.721	0.833
0.368	1.000	0.408	0.457	0.358	0.333
0.692	0.867	0.913	0.883	0.949	0.921
0.385	1.000	0.515	0.532	0.449	0.615
0.684	0.714	0.779	0.810	0.769	0.667
0.833	1.000	0.909	0.911	0.935	0.911
0.065	0.362	0.131	0.182	0.169	0.185
0.686	1.000	0.810	0.788	0.800	0.908
0.724	0.800	0.771	0.746	0.795	0.793
0.483	0.840	0.669	0.634	0.680	0.768
0.765	0.852	0.849	0.837	0.852	0.885
0.821	0.867	0.832	0.835	0.808	0.877
0.719	1.000	0.840	0.838	0.815	0.897
0.719	0.804	0.752	0.710	0.799	0.782
0.531	0.846	0.712	0.695	0.707	0.774
0.789	0.857	0.861	0.839	0.870	0.914
0.806	0.870	0.818	0.808	0.818	0.846
0.826	1.000	0.940	0.925	0.958	0.944
0.565	1.000	0.506	0.522	0.464	0.545
0.875	1.000	0.847	0.938	0.744	0.714
0.905	1.000	0.934	0.917	0.945	0.951
0.800	0.931	0.868	0.880	0.950	0.950
0.652	0.742	0.676	0.747	0.764	0.833
0.214	0.265	0.373	0.382	0.333	0.434
0.652	0.667	0.756	0.747	0.782	0.727
0.857	0.917	0.920	0.881	0.952	0.959
0.619	0.769	0.759	0.726	0.780	0.805
