# Supplementary figures and images for: Functional Biogeography as Evidence of Gene Transfer in Hypersaline Microbial Communities
Source: PLoS One. 2010 Sep 23;5(9):e12919. doi: 10.1371/journal.pone.0012919 (PMC2950788; doi:10.1371/journal.pone.0012919)

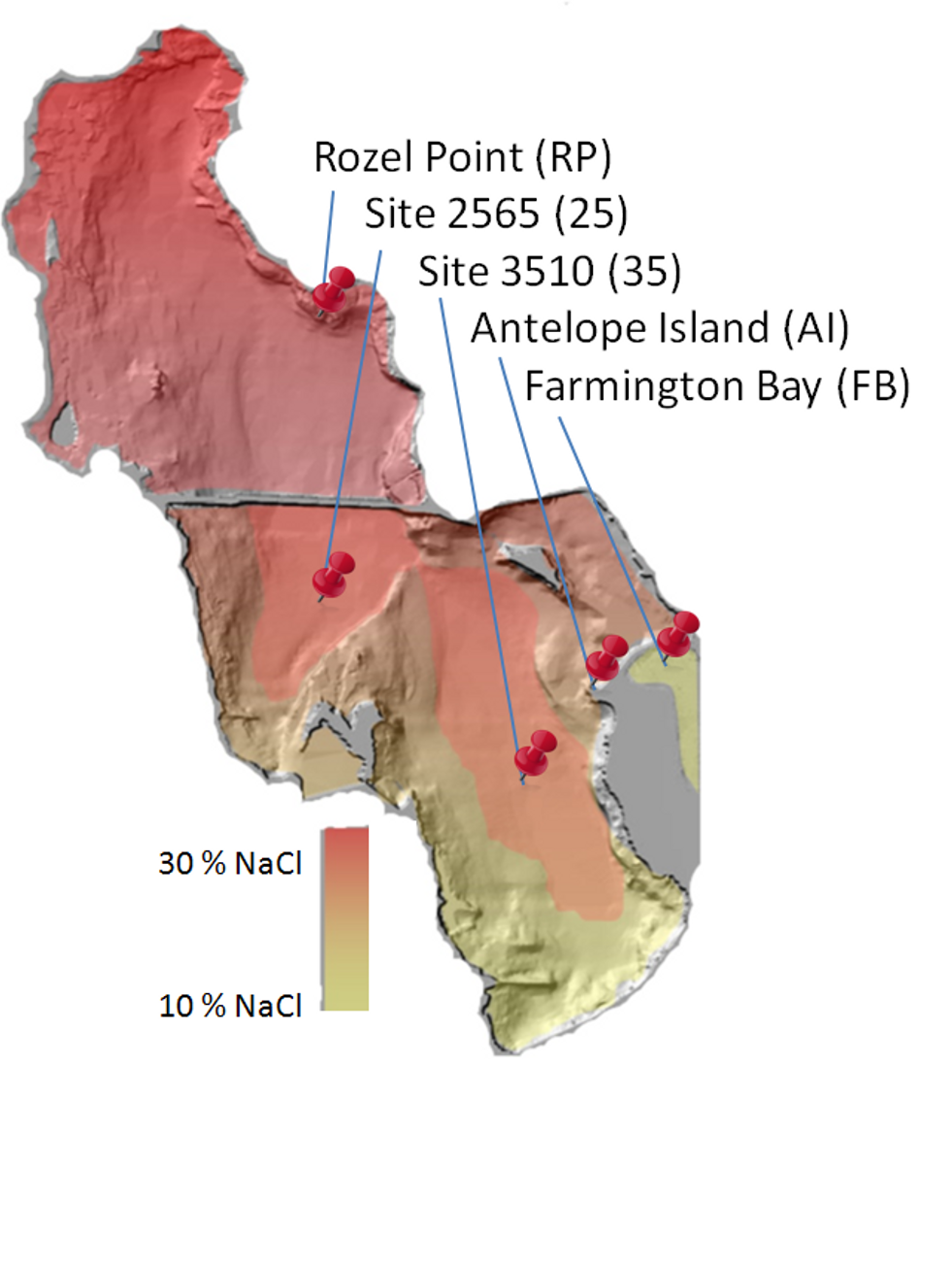

Supplement: Figure S1 — Sample locations along the salinity gradient in Great Salt Lake. Sample sites 3510 and 2565 are USGS collection sites and samples were collected at the surface, deep brine layer, and the interface between surface and deep brine. (1.01 MB TIF) [file pone.0012919.s003.tif]

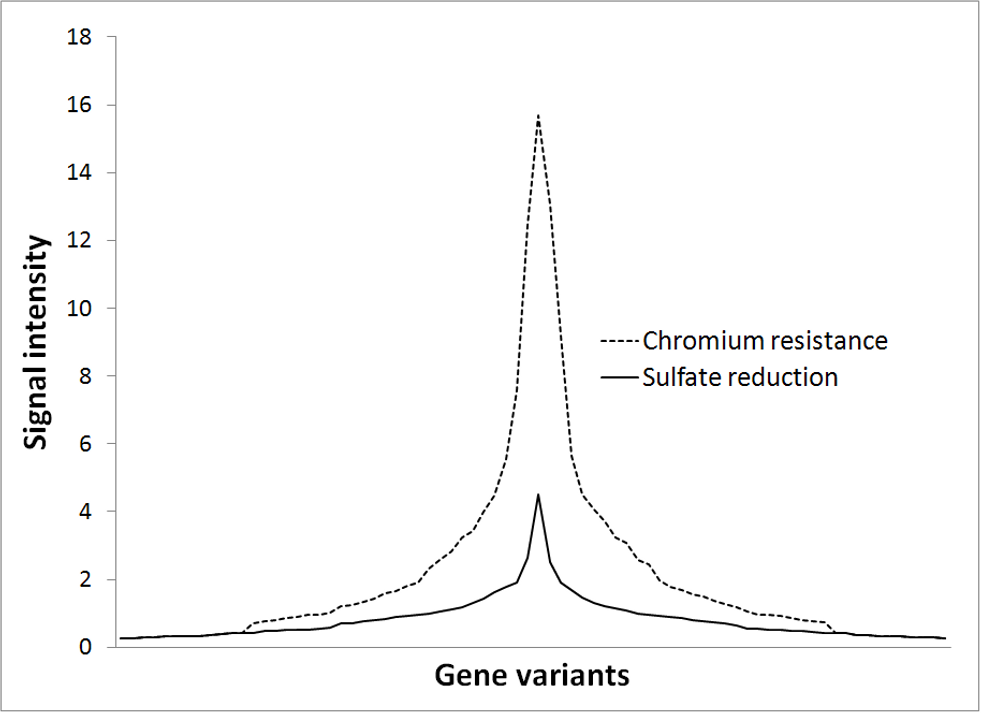

Supplement: Figure S2 — Example of curves from functional genes in 3510 surface sample used to determine selective pressure. (0.18 MB TIF) [file pone.0012919.s004.tif]

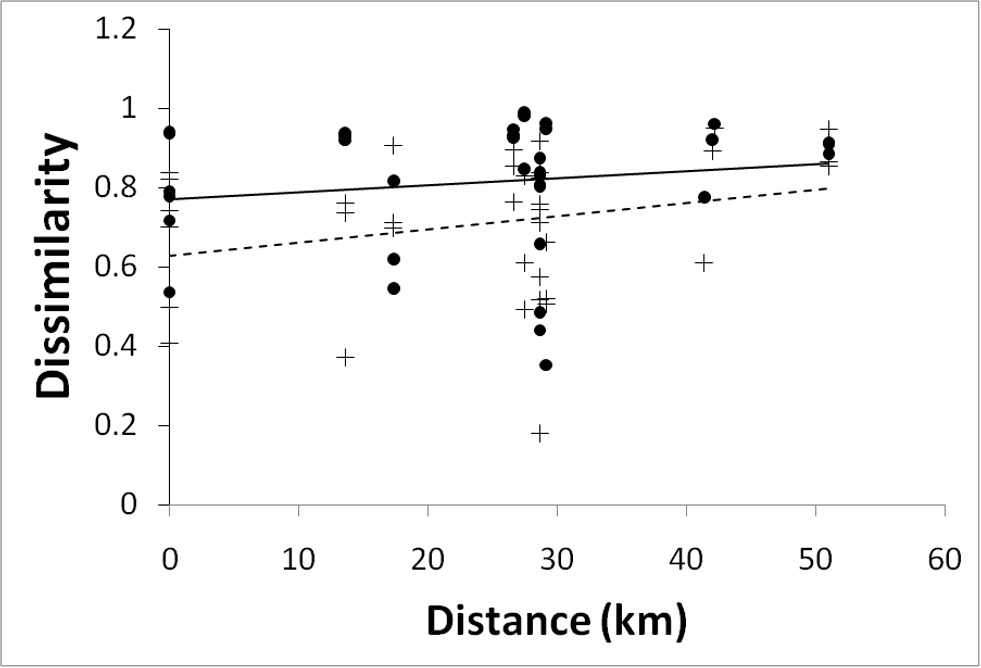

Supplement: Figure S3 — Weak correlation between dissimilarity and geographic distance. Circles represent taxonomic genes (solid line is linear regression). Cross hatches represent functional genes (dashed line is linear regression). (0.14 MB TIF) [file pone.0012919.s005.tif]

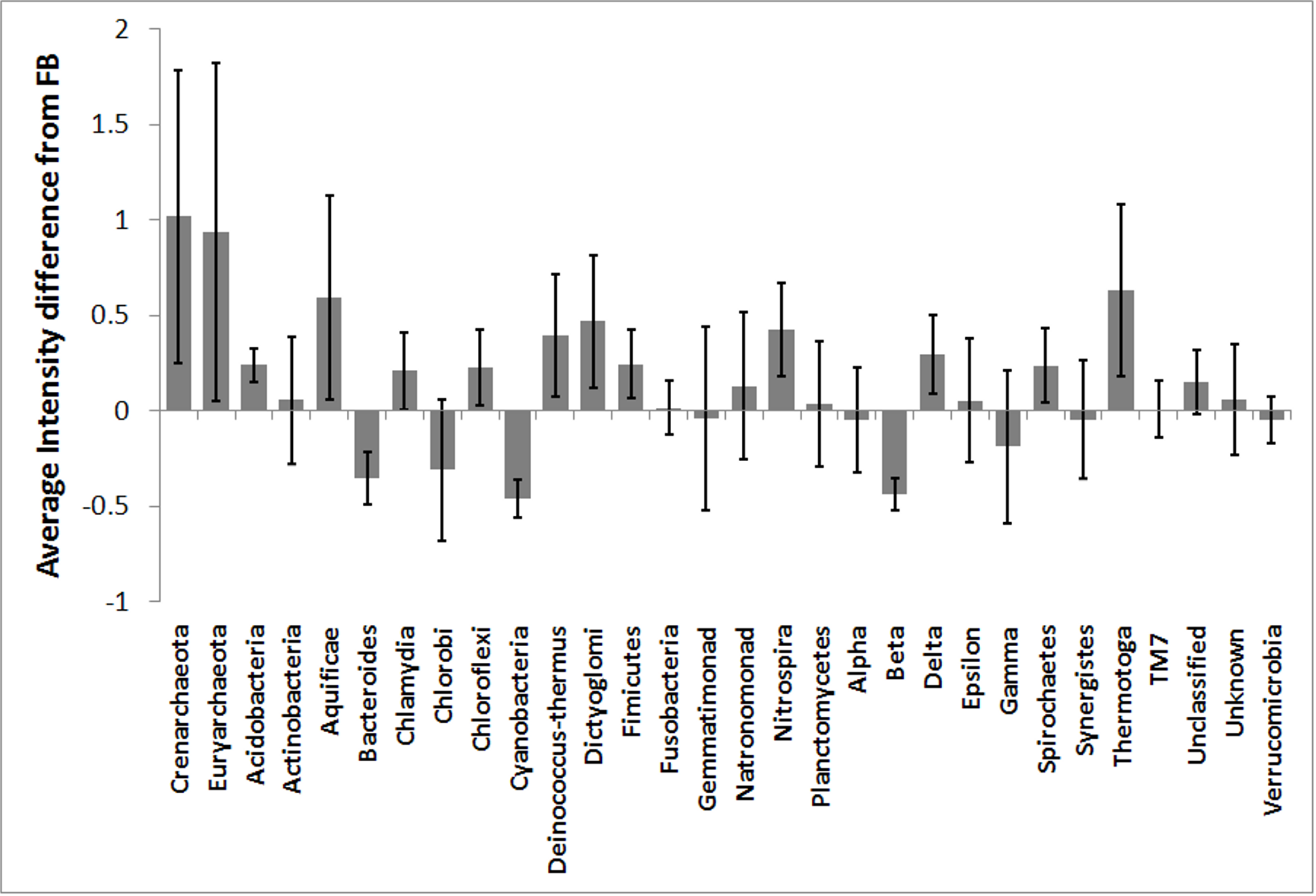

Supplement: Figure S4 — Major phylogenetic shifts due to increased salt. Farmington Bay (FB) was used as reference and the Log2 difference in intensity values are averaged (error = standard deviation) to indicate significant shifts due to high salt. (0.11 MB TIF) [file pone.0012919.s006.tif]

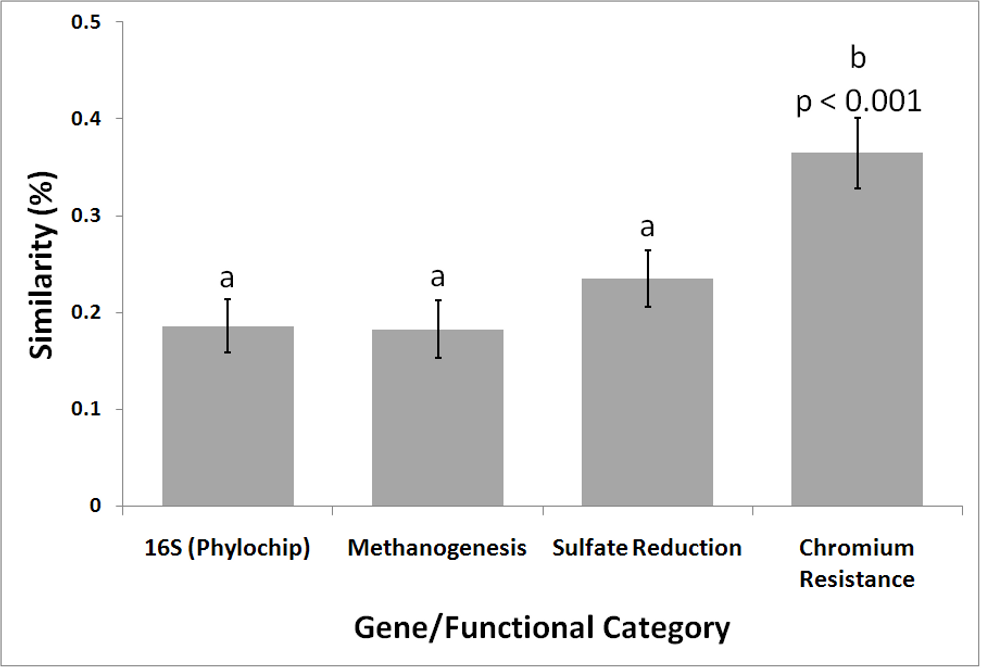

Supplement: Figure S5 — Average similarity of different genes throughout Great Salt Lake. 16S rDNA (phylochip)gene similarity is not significantly different from taxonomic-dependent methane generation (GeoChip). Sulfate reduction (low selective pressure) is not significantly different in lake-wide similarity from taxonomic genes. Chromium (high selective pressure) biogeographic patterns are significantly different, suggesting independence from taxonomy (t-test). (0.23 MB TIF) [file pone.0012919.s007.tif]
